# Free radicals and ultrafine particulate emissions from the co-pyrolysis of *Croton megalocarpus* biodiesel and fossil diesel

**DOI:** 10.1186/s13065-018-0458-6

**Published:** 2018-08-07

**Authors:** Joshua K. Kibet, Bornes C. Mosonik, Vincent O. Nyamori, Silas M. Ngari

**Affiliations:** 1 Department of Chemistry, Egerton University, PO Box 536, Egerton, 20115 Kenya; 2 Department of Physical and Biological Sciences, Kabaraka University, Private Bag, Kabarak, Kenya; 3 School of Chemistry and Physics, University of KwaZulu-Natal, Westville Campus, Private Bag X54001, Durban, 4000 South Africa

**Keywords:** Biodiesel, Co-pyrolysis, Free radicals, Nanoparticulates

## Abstract

**Background:**

The atmosphere has become a major transport corridor for free radicals and particulate matter from combustion events. The motivation behind this study was to determine the nature of particulate emissions and surface bound radicals formed during the thermal degradation of diesel blends in order to assess the health and environmental hazards of binary transport fuels.

**Methodology:**

Accordingly, this contribution explored the interactions that occur when *Croton megalocarpus* biodiesel and fossil diesel in the ratio of 1:1 by weight were co-pyrolyzed in a quartz reactor at a residence time of 0.5 s under an inert flow of nitrogen at 600 °C. The surface morphology of the thermal char formed were imaged using a Feld emission gun scanning electron microscope (FEG SEM) while Electron paramagnetic resonance spectrometer (EPR) was used to explore the presence of free radicals on the surface of thermal char. Molecular functional groups adsorbed on the surface of thermal char were explored using Fourier transform infrared spectroscopy (FTIR).

**Results:**

FTIR spectrum showed that the major functional groups on the surface of the char were basically aromatic and some methylene groups. The particulate emissions detected in this work were ultrafine (~ 32 nm). The particulates are consistent with the SEM images observed in this study. Electron paramagnetic resonance results gave a g-value of 2.0027 characteristic of carbon-based radicals of aromatic nature. Spectral peak-to-peak width (∆Hp-p) obtained was narrow (4.42 G).

**Conclusions:**

The free radicals identified as carbon-based are medically notorious and may be transported by various sizes of particulate matter on to the surface of the human lung which may trigger cancer and pulmonary diseases. The nanoparticulates determined in this work can precipitate severe biological health problems among humans and other natural ecosystems. 
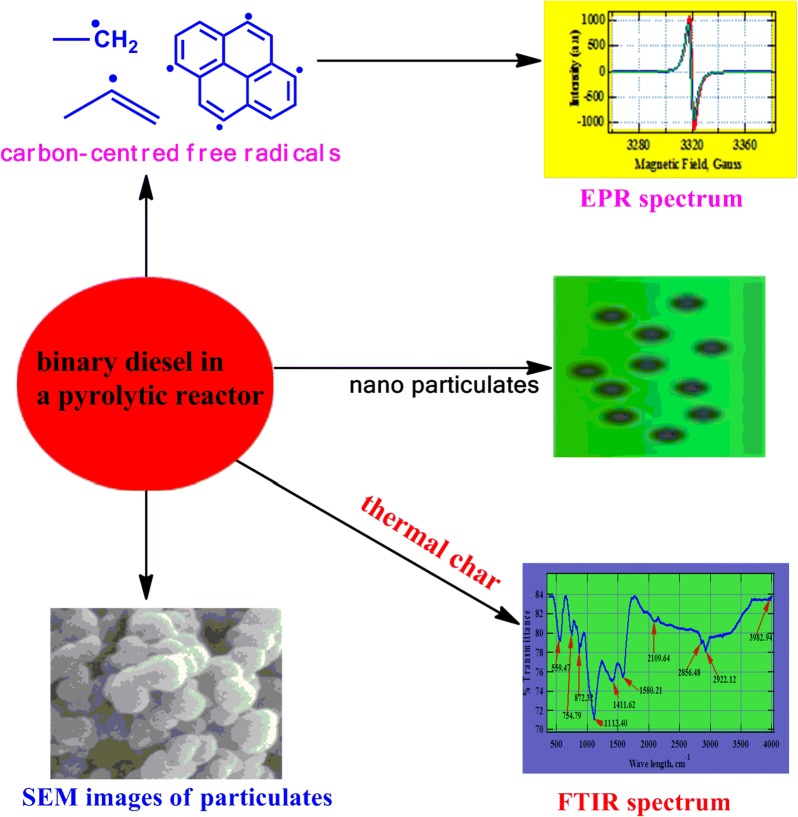

**Electronic supplementary material:**

The online version of this article (10.1186/s13065-018-0458-6) contains supplementary material, which is available to authorized users.

## Introduction

The atmosphere in general has become a major transport corridor for environmentally persistent free radicals and particulate pollution from combustion events. Consequently, environmental concerns in the use of petrol-based diesel has mounted urgent pressure towards clean energy combustion with a view to minimizing emission of toxic particulates from vehicular exhaust while embracing environmentally friendly transport fuels from biomass materials such as biofuels and model biodiesel–fossil diesel binary mixtures. The motivation behind this study is to explore the nature of particulates emitted and surface bound radicals formed during the thermal degradation of diesel blends in order to evaluate the health and environmental consequences of using binary transport fuels in combustion engines.

**Fig. 1:**
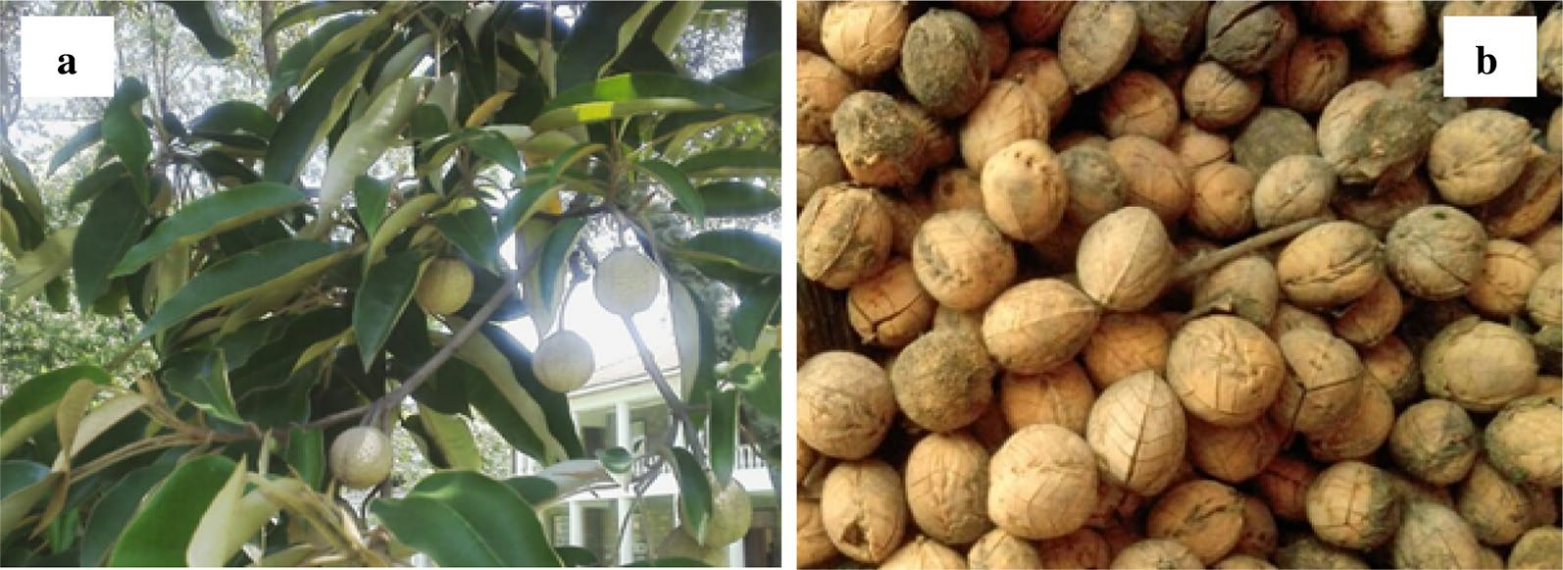
The *Croton megalocarpus* plant (**a**) and croton seeds (**b**) (photos taken by the author)

The major molecular components of biodiesel are mono-alkyl esters of fatty acids extracted from animal fat and vegetable oils such as *Croton megalocarpus* oil, canola oil and castor oil [1]. Of significant importance is the use of non-edible seed oil to produce biodiesel because they are not only economical but also does not interfere with the human food chain. Figure 1 shows the *C. megalocarpus* plant and its seeds which is the central source of the biofuel used in this study. Croton is one of the largest plant species of the *Euphorbiaceae* family and is well known for producing diverse uses ranging from medicinal, poultry feeds to processing of poison for use in hunting of game meat [2–4]. The plant is indigenous and is widely spread in the tropics especially East and Sub-Saharan Africa [5]. Recently, there has been pronounced research interest on the plant as a feasible biodiesel resource [6, 7]. Previous research surveys concluded that it has the highest raw oil production potential of 1.8 tones ha^−1^ year^−1^ compared with 1 tone ha^−1^ year^−1^ of *Jatropha curcas* [8]. The plant species of the croton genus seeds contain approximately 32% oil yield by weight [8].

On the other hand, diesel engines powered by fossil fuels are known to emit massive particulate matter, nitrogen oxides and greenhouse gases, hence there is need to develop cleaner energy transport fuels. In this respect, biodiesel has been shown to reduce environmental pollutants such particulate matter, carbon monoxide and unburned hydrocarbons [9]. Previous studies on biodiesel blends of ~ 20% indicated a reduction of about 15% in particulate matter emissions, carbon monoxide, total hydrocarbons, and other toxic molecular by-products of combustion such as aldehydes and polyaromatic hydrocarbons [10]. Therefore, it is understood that diesel blends of varying ratios (biodiesel and petroleum diesel) may optimize engine performance but the toxicity of emission by-products is something that needs to be probed exhaustively. In general, particulate emissions from combustion of transport fuels carry with them surface bound radicals that may have detrimental impacts on both human and environmental health. Pyrolysis experiments are indispensable in mimicking the actual reaction processes taking place inside the internal combustion engine.

The basic phenomena that occur during the thermal degradation of an organic sample is the initiation of pyrolysis reaction events which result in the evolution of organic volatiles and the formation of thermal char [11]. Pyrolysis therefore remains a central chemical process in the utilization of renewable energy, and generation of aromatic feed stocks [12, 13]. The main products from pyrolysis are organic volatiles, charcoal and gases, depending on the operating conditions such as temperature, nature of organic matrix, heating rate, residence time and engine design [14].

Co-pyrolysis of organic mixtures explores the possibility of reducing the formation and emission of toxic free radicals and particulates to the environment, as well as the existence of interactions between biodiesel materials and fossil fuels in the formation of thermal chars [15, 16]. Therefore, the study of interaction of biomass components and fossil materials in combustion systems with a view to optimizing engine performance is fundamental. Despite the availability of a plethora of data from individual pyrolysis of model compounds of biodiesel components (croton oil, sunflower, olive oil etc.) and fossil model materials such as coal and kerogen, the co-pyrolysis of biodiesel components and conventional diesel has received little attention hence co-pyrolysis studies of binary fuels (biofuel-fossil fuel) may have crucial leads towards achieving clean energy combustion. The primary emphasis of these studies is to determine the formation particulate emissions, nature of the resultant thermal char, and environmentally persistent free radicals for a thorough evaluation of binary transport fuels.

Environmentally persistent free radicals being one of the pollutants generated during the burning of fuels may be responsible for oxidative stress resulting in cardiopulmonary diseases and probably the exposure to airborne fine particles that are major precursors for malignant growth that ultimately lead to cancer [17]. Vehicular exhaust from combustion of gasoline, diesel, and other petroleum fuels is a dominant contributor of fine (PM_2.5_) and ultrafine (PM_0.1_) particulates and may contain emissions of carbonaceous particles with fused and free polycyclic aromatic hydrocarbons (PAHs) [18]. Furthermore, ambient PM is believed to contain persistent free radicals and reactive oxygen species (ROS) usually implicated in cellular damage and initiation of chronic pulmonary diseases [19, 20]. Persistent free radicals contribute to decreased lung function, promotion of asthma, bronchitis, and pneumonia, especially in children residing in areas of high levels of particulate pollution [21]. Although exposure to PM_0.1_ has been linked to diminished lung health, the underlying biological mechanisms responsible for enhanced exposure remain undefined [22]. Previous studies have also shown that women exposed to high levels of PM_10_, especially those containing surface bound radicals have given birth to children with small heads, and small bodies, and this has been known to impact negatively on their cognitive skills in addition to being vulnerable to carcinogens and mutagens [23]. It is against this evidence that the study of particulate emissions from model transport fuels has become important.

Although inventories on the pyrolysis of pure biodiesel and pure petroleum diesel are available in literature, very little information is known on the co-pyrolysis of binary mixtures of biodiesel and petroleum diesel. Nonetheless, some studies have explored binary blends in the range of 10–41% by weight and observed a reduction in particulate emissions with respect to NOx and polyaromatic hydrocarbons (PAHs) [6]. Binary diesel blends are predicted to achieve optimum engine efficiency. Accordingly, this investigation restricts itself to typical high temperature combustion of a heat engine (600 °C) and equimolar (by weight) mixtures of biodiesel and fossil diesel as model engine loads during combustion. This investigation will discuss extensively the particulate pollution, the nature of thermal char and surface bound radicals from the co-pyrolysis of *C. megalocarpus* biodiesel, and petroleum based diesel believed to have serious implications on both the physical and the biological environments.

## Methodology and materials

### Materials

All chemicals and reagents used in this study were of analytical grade and were purchased from Sigma Aldrich Inc., (St. Louis Missouri, USA) through its subsidiary, Kobian Kenya, Ltd. Croton oil was prepared by solvent extraction using hexane before it was converted to biodiesel through trans-esterification process and eventually subjected to American Society for Testing and Materials (ASTM) D 6751 standards [24]. The details of laboratory preparation of croton biodiesel are reported elsewhere in literature [25]. Commercial diesel was purchased from a local out let and used without further treatment. A muffle heating furnace with a temperature range ≈ 20–1000 °C was purchased from Thermo-Scientific Inc., USA. The reactor was fabricated in our laboratory by a glass blower while nitrogen of ultrahigh purity ≥ 99.99% (grade 5.0) was purchased from BOC gases, Kenya.

### Co-pyrolysis of *C. megalocarpus* biodiesel and petroleum diesel

In order to investigate the nature of particulate emissions and formation of environmentally persistent free radicals for optimization of clean energy combustion, binary mixtures in the ratio of 1:1 by weight were introduced into a pyrolysis reactor. Accordingly, 5 mg of *C. megalocarpus* biodiesel and 5 mg of petroleum diesel were mixed and placed in a quartz reactor of volume ≈ 7.85 cm^3^ housed in a muffle furnace (Thermo-Scientific Inc., USA). Pyrolysis was conducted at 600 °C under a flow of nitrogen at a residence time of 0.5 s at 1 atmosphere. Five replicates were conducted in this experiment. The residence time was determined from the conventional ideal gas formula (Eq. 1).

1 $$t_{0} = \left( {\frac{{\pi r^{2} L}}{{F_{0} }}} \right)\left( {\frac{{T_{1} }}{{T_{0} }}} \right)x\left[ {1 + \frac{{P_{d} }}{{P_{0} }}} \right]$$

where *t*_0_ is the residence time, *F*_0_ flow rate of the pyrolysis gas and *P*_*d*_ is the pressure difference between the inlet pressure and the pressure inside the reactor. Ideally the pressure difference is 0 because the ambient pressure and the reactor pressure are supposedly similar ~ 1 atm. while *T*, *L* and *r* represent the temperature, length of the reactor, and the radius of the tubular reactor respectively. The subscript _**0**_ denote original parameters (ambient) while the subscript _**1**_ denotes the parameters inside the reactor.

### Electron paramagnetic spectroscopy (EPR)

About 5 mg thermal char sample from the co-pyrolysis of biodiesel and commercial diesel was analyzed using a Bruker EMX-20/2.7 EPR spectrometer (X-band) with dual cavities, modulation and microwave frequencies of 100 kHz and 9.516 GHz, respectively [26, 27]. The typical parameters were: sweep width of 200 G, EPR microwave power of 1–20 mW, and modulation amplitude of ≤ 6 G. Time constant and sweep time were 16 s and 84 s, respectively. The value of the g factors was calculated using Bruker’s WINEPR program, which is a comprehensive line of software that allows control of the Bruker EPR spectrometer, data-acquisition, automation routines, tuning, and calibration programs on a windows-based personal computer [28]. The actual g-value for the spectrum was estimated by comparison with a 2,2-diphenyl-1-picrylhydrazyl (DPPH) standard.

### Scanning electron microscopy (SEM) analysis

Approximately 5 mg of thermal char was introduced into 1 mL methanol and gold grids dipped into the prepared thermal char sample. A Twister was used to pick the gold grids from the char sample. The sample was stuck to aluminium SEM stubs with carbon tape. These were subsequently gold coated in a Quorum Q150 RES sputter coater [29]. The grids were allowed to dry in air before putting them into the analysis chamber of a Zeiss Ultra Plus (Germany) field emission gun scanning electron microscope (FEG SEM) [30]. For enhanced image clarity, a second sample of char was coated with a 3 nm Au layer to allow for higher resolution images to be obtained. All images were taken at an angle of 45° to increase the definition of the surface morphology [31]. The SEM machine was then switched on and imaging of the sample conducted at 20.0 kV using a light emitting diode (LED). The lens was varied at various resolutions to obtain a clear focus of the sample image. A detailed procedure for SEM analysis is reported elsewhere [29, 31]. Additional images collected from this study are reported in the Additional file 1: Figure S3.

**Fig. 2:**
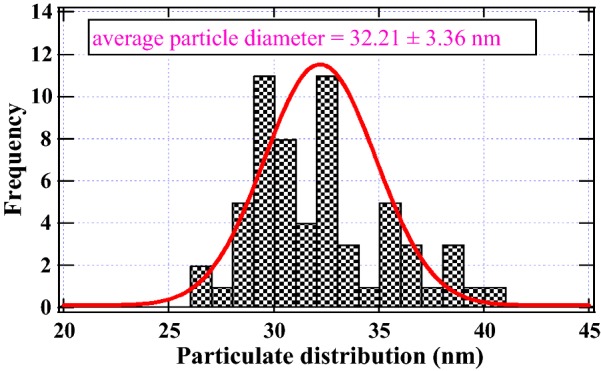
Particulate size distribution for the co-pyrolysis of croton biodiesel and fossil diesel

Image *J* computational code was used to determine the particulate size of the thermal char and a distribution curve of particulate size was then extracted using *Igor* graphing software (Igor ver. 5.0). The mean sizes of the char particulates at 600 °C was reported and presented as a Gaussian curve in which the peak of the curve gave the mean of the thermal particulates. Four SEM micrographs were used to extract the particulate size data for drawing the Gaussian curve presented in Fig. 2.

### Fourier transform infrared (FTIR) spectroscopy

Conventionally, absorption spectra were collected using an Agilent FTS 7000e FTIR bench top spectrometer equipped with a liquid nitrogen-cooled mercury cadmium telluride detector and a heated (65.1 °C) seven-reflection diamond ATR crystal (Concentrate IR, Harrick Scientific Products, Pleasantville, NY) described elsewhere in literature [32]. Attenuated total reflection Fourier-transform infrared (ATR-FTIR) spectroscopy was used in this investigation. ATR-FTIR spectra (256 co-added scans) were collected at the 4 cm^−1^ resolution over the wave number range of 4000–500 cm^−1^ at an average of 4 scans [33, 34]. FTIR is one of the most important and versatile analytical techniques available to the current crop of scientist [35]. FTIR spectrum of the control (blank) sample was run, Additional file 1: Figure S2.

## Results and discussion

In this work, unique data on the co-pyrolysis of equimolar mixture of croton biodiesel and fossil diesel is presented. The central point of this investigation is the analysis of thermal char from a spectroscopic perspective. Of fundamental focus are the free radicals immobilized on the surface of particulate emissions suspected to be architects of a number of health and environmental problems. It is well known in the combustion community that the shorter the residence time, the smaller the particulate emissions because shorter residence times discourage agglomeration in particle formation—particle recombination time is too short [28].

## Co-pyrolysis of *C. megalocarpus* biodiesel and fossil diesel

The term particulate matter refers to particle pollution—a matrix of aerosol droplets, dust, smoke and soot of varying particulate sizes that pose serious health concerns [36]. The particle emissions presented in this study from the co-pyrolysis of the binary mixture of croton biodiesel and fossil diesel are classified as ultrafine particulates (~ 32 nm), the equivalent of 0.03 µm (cf. Fig. 2). These findings are disturbing from an environmental and a health perspective.

This study has shown that the thermal degradation of a mixture *C. megalocarpus* oil and fossil diesel gives rise to particulate fractions far much less than PM_0.1_ and are therefore considered the most damaging of all PM particulates because they may be inhaled deeper into the lung tissues thereby causing grave damage to both humans and other organisms. Wu and his co-workers [4] proposed the strictest of emission standards to be observed when designing the combustion engine that runs on biofuels owing to ultrafine particulates associated with its combustion [4]. However, it is not clear in their study why ultrafine particulate emissions were not investigated. As a general rule, the formation of emission particle formation in combustion systems proceeds via homogeneous nucleation (particulates < 100 nm) and agglomeration (particulates > 900 nm) processes [4, 37]. In this proposition, emission particles stick together to form chain-like structures and may contain surface bound radicals [37] considered injurious to the biological environment.

**Fig. 3:**
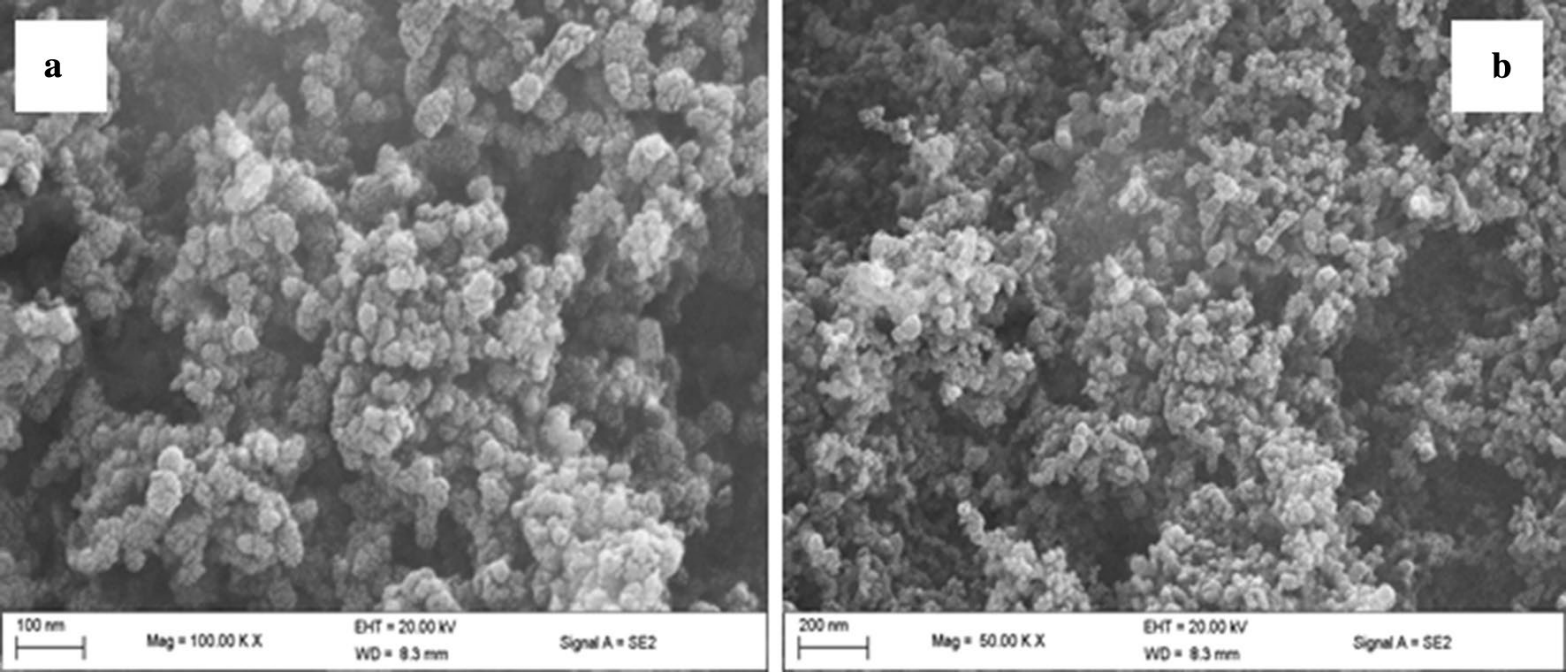
SEM image of biodiesel–fossil diesel at an associated magnification of ×50,000 at 200 nm (**a**) and a magnification of ×100,000 at 100 nm (**b**)

Scanning electron micrographs from which the particulate size presented in this work was derived are presented in Fig. 3. Figure 3a was scanned at a magnification of 100,000× while Fig. 3b was imaged at a magnification of 50,000×. Clearly, the particulate matter identified in this study is far much less than PM_0.1_. The particulate sizes from the thermal char resulting from the co-pyrolysis of croton biodiesel and commercial diesel were estimated from several SEM micrographs in order to obtain sufficient data for the generation the distribution curve presented in Fig. 2. Image J computer software has robust proficiencies of computing the size distribution as well averaging particulates from SEM images. Additional micrographs at various magnifications are reported in the Additional file 1: Figure S3.

### Electron paramagnetic resonance spectroscopy

**Fig. 4:**
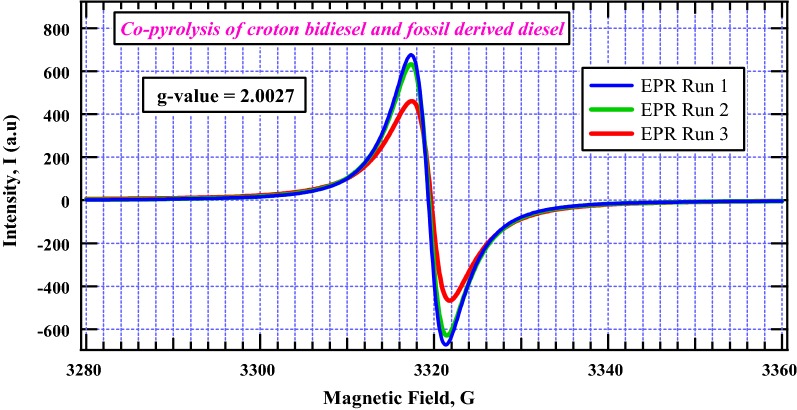
Diesel blend thermal char EPR spectra—radical intensity as a function of magnetic field (EPR spectra showing intensity as a function of g-value are reported in Additional file 1: Figure S1)

The g-value of the free radicals in thermal char was found to be 2.0027 which can be considered as pure carbon-based radicals because they are significantly close to that of a free electron, 2.0023 (one of the most accurate conventional constants ever known in physics). The peak-to-peak width of the EPR signal was quite narrow, (4.42 G). The EPR spectrum of the thermal char had a strong anisotropic singlet peak at around 3320 G (cf. Fig. 4). The spin density for run 1 (conducted 20 days after the preparation of the thermal char) was found to be 9.18 × 10^19^ spins/g and 3.84 × 10^17^ spins/cm. The thermal char was monitored over a period of 80 days in order to investigate their stability. The EPR spectra for this study are presented in Fig. 4. For clarity, the EPR signal for run 4 is not plotted in Fig. 4. However, plots of g-values as a function of magnetic field for selected EPR runs (including run 4) are reported in the Additional file 1: Figure S1.

EPR run 3 was significantly broad and had a lower intensity while runs 1 and 2 were symmetrical and quite intense. This broad feature in run 3 may be attributed to the break down in the Heisenberg exchange interaction. The EPR parameters for the thermal char explored in this work are presented in Table 1. Evidently from Table 1, the thermal char had fairly high spin densities. Even after 80 days, the spin density (spins/g) in thermal char had decreased only by about 15% of the initial run conducted 20 days after the co-pyrolysis experiment. This decrease is also consistent with that realized for spins/cm over a similar period of time (~ 14%). These observations demonstrate that the free radicals bound on the surface of thermal char are, indeed, very stable and are can thus be accurately classified as environmentally persistent free radicals (EPRs).

**Table 1:** The EPR parameters for the thermal char formed from the co-pyrolysis of the binary mixture of biodiesel and conventional diesel

Char/run	Time (days)	g/cm	spins/cm	spins/g
BCD (1)	20	0.0022	3.84 × 10^17^	9.18 × 10^19^
BCD (2)	50	0.0022	3.63 × 10^17^	8.03 × 10^19^
BCD (3)	60	0.0022	3.55 × 10^17^	7.88 × 10^19^
BCD (4)	80	0.0022	3.34 × 10^17^	7.81 × 10^19^

*BCD* binary mixture of biodiesel and conventional diesel

### FTIR features of the thermal char

**Fig. 5:**
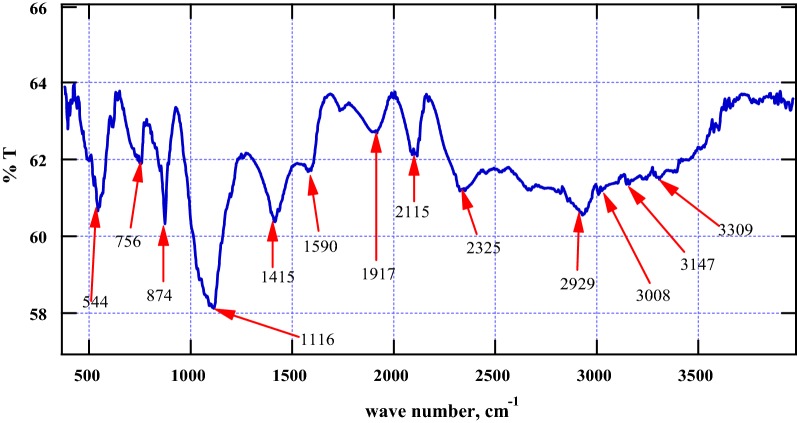
FTIR absorption bands for the char formed from the co-pyrolysis of croton biodiesel and conventional diesel (FTIR absorption spectrum for the blank is reported in Additional file 1: Figure S2)

The investigation of the surface functional groups of the thermal char from the co-pyrolysis of the binary mixture—croton biodiesel and fossil diesel using FTIR gave several principal bands as shown in Fig. 5. The intense broad absorption peak δ_s_ (1116 cm^−1^) is associated with in-plane bending of –CH_3_ in the possible aromatic structure of thermal char. The absorption bands υ_sa_ (2929 cm^−1^) and υ_s_ (3008 cm^−1^) are characteristic of asymmetrical and symmetrical stretching of methylene (–CH_2_–) groups for long chain aliphatic hydrocarbons. The sharp vibration υ_s_ (2115 cm^−1^) may be attributed to a C≡C (alkyne) which could be present in the thermal char. The absorption peak υ_s_ (2325 cm^−1^) is probably a nitrile (–C≡N) that could be bonded to the char matrix. Moreover, the moderately weak absorption band υ_s_ (1590 cm^−1^) is an aromatic C–C double bond while δ_s_ (1415 cm^−1^) can judiciously be assigned to –CH_2_ bending modes in arenes.

The absorption bands at 874 and 756 cm^−1^ are consistent with δ(–CH_2_) signature vibrations for in-plane and out of plane bending modes in aromatic compounds, respectively. A sharp band appearing around 544 cm^−1^ may correspond to in-plane bending of the O=C–N group which could be present in the thermal char. All the surface functionalized groups identified in this investigation suggest that the thermal char is aromatic. Additionally, the stability of free radicals explored in this work as carbon-centred ones may be delocalized within a highly conjugated π–π system [25].

### The health and environmental concerns

This investigation has demonstrated that the co-pyrolysis of croton biodiesel and petroleum based diesel gives rise to ultrafine particles in the nano region (~ 32 nm) which may contain surface immobilized radicals, and if inhaled may precipitate serious health implications. For instance, it has been established that animal studies on rats exposed to particulate nanoparticles of ~ 22 nm diameter have found their way into the connective tissue of the heart such as the fibroblast [35] and ultimately causing grave biological damage and cardiopulmonary death. Moreover, there is compelling evidence that within half an hour of exposure, large quantities of intra-tracheal implanted nanoparticles of ~ 20 nm diameters have been found in platelets in the pulmonary capillaries of rats [37]. Additionally, the findings on free radicals bound to nanoparticles from this investigation are very disturbing because they are precursors for severe environmental and health problems.

Clearly, in the search of alternative transport fuels such as binary diesel fuels explored in this work, the question of ultrafine emissions that carry with them surface bound radicals is of grave health concern. These particulates are extremely hazardous especially because they can be inhaled deeper and possibly find their way into the blood stream and thus may be carried into the heart during the blood circulation processes. Nanoparticles therefore are progenitors for fatal injury in biological cells and may trigger the production of reactive oxygen species (ROS) and ultimately cause oxidative stress, cardiac diseases, and even body mass waste. Thus nanoparticulate emissions detected in this work may somewhat suggest an impediment in the search for environmentally friendly transport fuels. Nonetheless, engine designs fitted with efficient catalytic chambers and precipitators can impede the emission of ultrafine particulate, and probably improve the efficiency of binary diesel blends in motor systems.

**Scheme 1:**
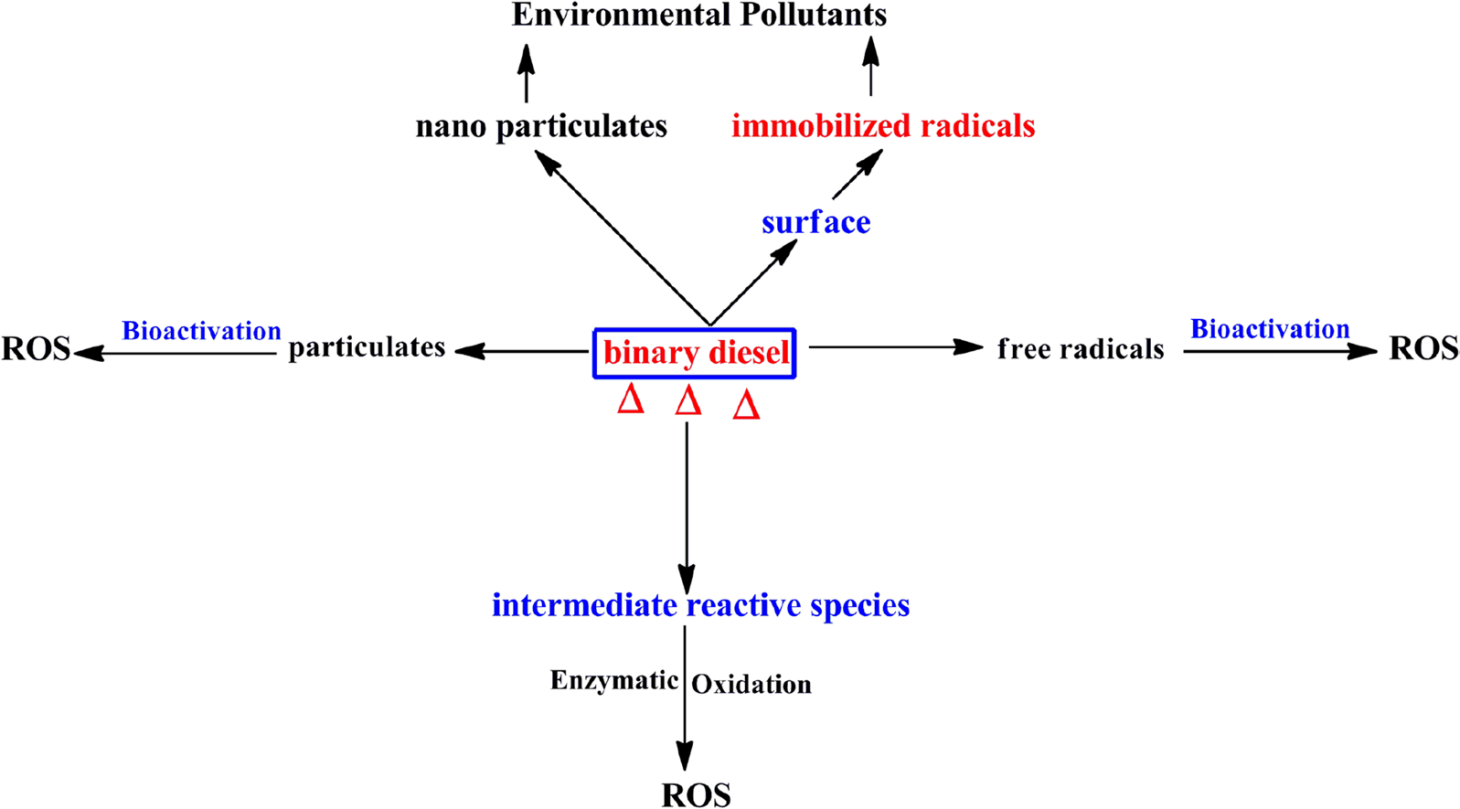
Mechanistic channels showing the generation of toxic species from the pyrolysis of binary diesel (up-pointing triangle indicates pyrolysis) and their predictive effects on the biological and the environmental systems

The mechanistic processes culminating into the biological health and environmental health problems derived from this study are summarized in Scheme 1. While intermediate reactive species (molecular reactive species) have not been explored in this work, we believe they are central sources of free radicals and are therefore equally hazardous as particulate emissions and surface bound radicals. Bioactivation and enzymatic activation are the fundamental processes which occur when reactive species interact with biological systems to cause diseases such as cancers and pulmonary ailments [38]. These processes precipitate the formation of reactive oxygen species (ROS) responsible for various medical problems suffered by man and other ecosystems.

Remarkably, toxicological and epidemiological studies have shown that exposure to combustion particulate emissions, especially those carrying with them surface bound radicals as is the case in this study are well known precursors for such ailments as, lightheadedness, chronic respiratory problems, cardiopulmonary death, asthma and cancers [39]. Other studies have also established that particulates can encourage inheritable diseases such as leukemia [40]. Besides health concerns, particulate emissions are known to combine with other air pollutants to form atmospheric brown clouds which are exceptional progenitors for numerous adverse environmental problems afflicting humans and other living organisms [17]. The fact that previous studies proposed stringent emission standards to be applied when designing biofuel based engines is a grave concern in the search for alternative fuels [4]. This is highly consistent with the findings advanced in this work.

## Conclusions

This study has established that particulate emissions from the pyrolysis of a binary mixture of croton biodiesel and petrol-based diesel are ultrafine (~ 32 nm) and may be inhaled deeper into biological tissues, possibly finding their way into the red blood cells, alveoli, and the fibroblasts of the heart. The consequences of inhaling such particulates range from cell mutation, carcinogenesis, chronic coughs and cardiovascular death. Moreover, particulate emissions from the co-pyrolysis of croton biofuel and petroleum-based diesel carry with them surface bound radicals that may be of serious concern to both the biological and the physical environment. The free radicals identified in this study are carbon-based which may certainly be inhaled into the surface of the lungs being transported along by various sizes of particulate matter (PM) and are capable of causing pulmonary diseases, oxidation stress and cell aberrations. Based on the findings of this study it may be necessary to explore varying ratios of biofuels and conventional diesel in order to derive optimum working conditions of an internal combustion engine without compromising public and environmental safety.

## Additional file

**Additional file 1: Figure S1.** The diesel blend thermal char EPR spectra for runs 1 and 4; g-factor as a function of magnetic field. **Figure S2.** FTIR spectrum for the blank. **Figure S3.** SEM images of thermal char at various magnifications.
