# Supplementary material for: Free radicals and ultrafine particulate emissions from the co-pyrolysis of Croton megalocarpus biodiesel and fossil diesel
Source: Chem Cent J. 2018 Aug 7;12:89. doi: 10.1186/s13065-018-0458-6 (PMC6081488; doi:10.1186/s13065-018-0458-6)
Supplement: Supplementary file 1 — Additional file 1: Figure S1. The diesel blend thermal char EPR spectra for runs 1 and 4; g-factor as a function of magnetic field. Figure S2. FTIR spectrum for the blank. Figure S3. SEM images of thermal char at various magnifications. [file 13065_2018_458_MOESM1_ESM.docx]

**Free radicals and ultrafine particulate emissions from the co-pyrolysis of Croton *megalocarpus* biodiesel and fossil diesel**

Joshua K. Kibet*^1^, Bornes C. Mosonik^1,2^, Vincent O. Nyamori^3^ and Silas M. Ngari^1^

*author for correspondence: [jkibet@egerton.ac.ke](mailto:jkibet@egerton.ac.ke) Tel: +254 720 352 437

^1^Department of Chemistry, Egerton University, PO Box 536 -20115, Egerton, Kenya

^2^Department of Physical and Biological sciences, Kabaraka University, Private Bag, Kabarak

^3^School of Chemistry and Physics, University of KwaZulu-Natal, Westville Campus, Private Bag X54001, Durban 4000, South Africa

**Additional file 1**

The material presented here are important in enhancing the understanding of the concepts reported in the article. The Figures presented in the article were done using Igor graphing software (ver. 5.0).

**Figure S1:** The diesel blend thermal char EPR spectra for runs 1 and 4; g-factor as a function of magnetic field

**
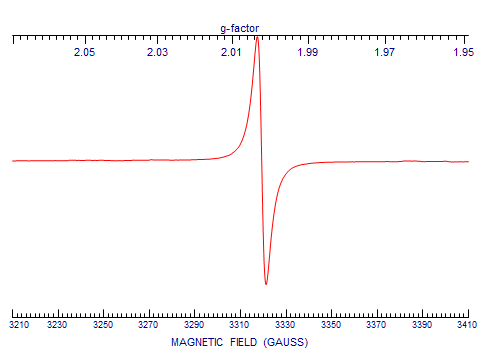
**

1. EPR spectrum for run 1

**
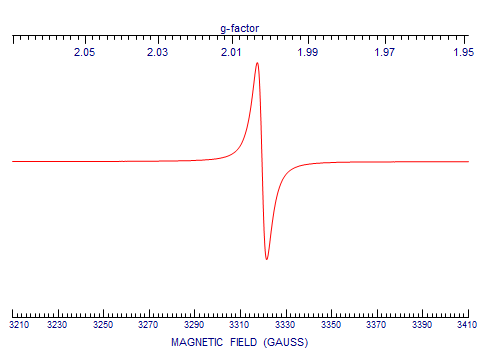
**

1. EPR spectrum for run 4

**Figure S2:** FTIR spectrum for the blank

**Figure S3.** SEM images of thermal char at various magnifications

**

**

1. SEM image at an associated magnification of X10,000 for the thermal char





1. SEM image at an associated magnification of X20,000 for the thermal char
